# Editorial: Daily challenges around physical exercise, nutrition and medication in type 1 diabetes

**DOI:** 10.3389/fendo.2023.1259535

**Published:** 2023-07-31

**Authors:** Othmar Moser, Klemen Dovc

**Affiliations:** ^1^ Exercise Physiology and Metabolism, Institute of Sports Science, University of Bayreuth, Bayreuth, Germany; ^2^ Division of Endocrinology and Diabetology, Department of Internal Medicine, Medical University of Graz, Graz, Austria; ^3^ Interdisciplinary Metabolic Medicine Trials Unit, Medical University of Graz, Graz, Austria; ^4^ Department of Pediatric Endocrinology, Diabetes and Metabolic Diseases, University Children’s Hospital, Ljubljana, Slovenia; ^5^ Faculty of Medicine, University of Ljubljana, Ljubljana, Slovenia

**Keywords:** exercise, type 1 diabetes, insulin, automatic insulin delivery, continuous glucose monitoring

Type 1 diabetes is characterized by autoimmune destruction of insulin-secreting pancreatic β-cells leading to disturbed glucose regulation and overt hyperglycemia. Consequently, individuals with type 1 diabetes have a lifelong need for insulin replacement therapy. The primary goal in the long-term care of type 1 diabetes is to maintain glucose levels physiologically as possible, mitigating the risk of micro- and macrovascular complications, which combined represent the primary cause of morbidity and mortality in developed societies (1–4). Recent advancements in diabetes technologies have revolutionized disease management, with (semi)automated and glucose-responsive treatment modalities allowing individuals to optimize glycemia, improve quality of life, and reduce the burden of type 1 diabetes. Furthermore, there has also been reported a global increase in uptake into everyday clinical practice (5–10). Alongside insulin replacement therapy, physical activity and exercise combined with a well-balanced diet are cornerstones in the management of diabetes. In numerous studies, it was demonstrated that regular physical activity improves glycemic outcomes and the quality of life compared to a sedentary lifestyle (11–15). Physical activity, especially structured exercise, has long been proposed as a major hurdle for automatic insulin delivery systems, and studies are now looking into how these systems are performing around physical activity (16–19). Exercise duration, modality, relative and absolute intensity, and fitness capacity (among others) affect glucose homeostasis in people living with type 1 diabetes. Several strategies for insulin adjustments and additional carbohydrate consumption regarding physical activity and exercise have been suggested, and these recommendations should be individualized and tailored based on the above-mentioned factors and treatment modalities (20–25).

Due to the inhomogeneous nature of physical activity exercise, activity-related hormonal responses, and different gender and age responses to physical activity, a personalized glucose management and nutritional plan should be followed.

This Research Topic of *Daily Challenges Around Physical Exercise, Nutrition and Medication in Type 1 Diabetes* covers diverse topics of both pre-clinical and clinical studies, addressing relevant questions surrounding the everyday decisions that individuals with type 1 diabetes encounter to overcome dysglycemia.

McCarthy et al. in their review “*The endocrine pancreas during exercise in people with and without type 1 diabetes: Beyond the beta-cell*” discussed the crucial role of the endocrine pancreas, beyond its role in digestion and metabolism at rest, in regulating energy during physical exercise. In type 1 diabetes, the destruction of β-cells leads to significant disruptions in glucose control; however, the impact of type 1 diabetes on other pancreatic hormones response during exercise and how they differ from individuals without type 1 diabetes are less investigated. Understanding these responses in both individuals with and without diabetes can help to identify clinically relevant adaptations for effective management of glucose levels around exercise, thus providing optimal clinical care through educated decision making. This knowledge is particularly relevant with the advancement of automated insulin delivery and emerging algorithms that detect physical activity, as it can bridge the gap between clinical and engineering considerations for automatic insulin delivery requirements during physical activity.

Barlovic et al. evaluated in their review “*Exercise and nutrition in type 1 diabetes: Insights from the FinnDiane cohort*” the main findings from the Finnish Diabetic Nephropathy Study (FinnDiane), a long-term research project involving over 8,000 individuals with type 1 diabetes over a period of 25 years. The authors discuss different topics from research in this well-studied cohort, including associations between physical activity, glycemic outcomes, chronic complications, and mortality or associations between different aspects of nutrition, glycemic outcomes, and chronic complications of type 1 diabetes. Importantly, this discussion of the FinnDiane project further underpins the importance of living a physically active lifestyle with type 1 diabetes.

Fitzpatrick et al. described in their paper “*Exercise, type 1 diabetes mellitus and blood glucose: The implications of exercise timing*” that the timing of exercise may play a crucial role in preventing glucose imbalances such as post-exercise hypo- or hyperglycemia. There is limited evidence summarizing the impact of exercise timing on glucose metabolism in type 1 diabetes; in this report, the authors suggest that resistance training or high-intensity interval training (HIIT) should be performed in the afternoon/evening when individuals are more likely to experience hypoglycemia since these types of exercise provide more glucose stability or even an increase in glucose levels. However, continuous aerobic exercise is recommended in the morning due to natural circadian elevations in blood glucose, providing additional protection against exercise-induced hypoglycemia. However, more well-designed studies are needed to further investigate the relationship between exercise timing and glycemic control, ultimately determining the most effective and safe exercise timing for individuals with type 1 diabetes.

Rilstone et al. provided in their study “*Nutritional support for a person with type 1 diabetes undertaking endurance swimming*” information about nutritional considerations for people with type 1 diabetes participating in long-distance open-water events, including personal testimony from a marathon swimmer with type 1 diabetes. Individuals with type 1 diabetes face additional complexities related to insulin management and manage it through high carbohydrate intake. This paper aims to provide insights and recommendations for individuals with type 1 diabetes engaging in long-distance swimming activities, highlighting the main considerations and suggestions for insulin management strategies.

Valder and Brinkmann in their opinion article “*Is intake of fruit juice useful in exercise-induced hypoglycemia prevention in individuals with type 1 diabetes mellitus?*” discussed the role of fruit juice in preventing exercise-induced hypoglycemia in individuals with type 1 diabetes and addressed associated health concerns. The authors provided some practical recommendations regarding nutrition, carbohydrate intake amount, and glucose monitoring before, during, and after physical activity. Very clinically relevant to note for people with type 1 diabetes, when fructose is consumed during exercise, this might further increase systemic lactate concentration, and the extent of blood glucose increase might be lower when compared to glucose consumption.

Vlcek et al. reported in their patient-led qualitative study “*How we do it: A qualitative study of strategies for adopting an exercise routine while living with type 1 diabetes*” the experiences of individuals living with type 1 diabetes on how they adopt and maintain an active lifestyle while managing the risks of hypoglycemia and glucose fluctuations. Semi-structured interviews and focus groups were conducted, and interpretive description analysis was used to identify themes and strategies associated with staying physically active with type 1 diabetes. In their study, the authors found that structure and organization, trial and error learning, psychosocial aspects, diabetes technology, education, and peer support were key facilitators of regular physical activity. Strategies to overcome barriers included utilizing technology, integrating psychosocial support, adjusting insulin and carbohydrate intake, and planning when and how to perform exercise. Their findings emphasize the importance of personalized approaches, understanding individual glycemic responses, and incorporating supportive tools and resources for individuals with type 1 diabetes to maintain an active lifestyle.

Gianini et al. demonstrated in their article “*Patient reported outcome measures in children and adolescents with type 1 diabetes using advanced hybrid closed loop insulin delivery*” that the use of advanced automatic insulin delivery systems resulted in decreased fear of hypoglycemia, less emotional distress, increased quality of life, and reduced burden of type 1 diabetes management together with improved metrics of glycemia. This was investigated by utilizing mixed methods research design using both quantitative and qualitative approaches.

Within the last article in our Research Topic, Zaharieva et al. in “*Adding glycemic and physical activity metrics to a multimodal algorithm-enabled decision-support tool for type 1 diabetes care: Keys to implementation and opportunities*” presented an overview of the essential steps of integrating exercise data into an algorithm-enabled patient prioritization and remote patient monitoring program. This care model integrates continuous glucose monitoring data to prioritize patients for weekly reviews by clinical diabetes, has improved clinical workflows, and has been associated with improved glucose outcomes in newly diagnosed young people with type 1 diabetes. Incorporating exercise data (such as step count or heart rate) into the current continuous glucose monitoring (CGM)-based care model could produce additionally clinically relevant information such as identifying whether individuals are meeting physical activity recommendations and would allow for personalized care and better-informed decisions around individual needs, insulin-dosing decisions, and overall diabetes management.

## Author contributions

OM: Writing – original draft, Writing – review & editing. KD: Writing – original draft, Writing – review & editing.
